# The role of RNA-modifying proteins in renal cell carcinoma

**DOI:** 10.1038/s41419-024-06479-y

**Published:** 2024-03-19

**Authors:** Muna A. Alhammadi, Khuloud Bajbouj, Iman M. Talaat, Rifat Hamoudi

**Affiliations:** 1 Research Institute of Medical and Health Sciences, University of Sharjah, Sharjah, P.O. Box 27272, United Arab Emirates; 2 Department of Clinical Sciences, College of Medicine, University of Sharjah, Sharjah, P.O. Box 27272, United Arab Emirates; 3 Department of Basic Sciences, College of Medicine, University of Sharjah, Sharjah, P.O. Box 27272, United Arab Emirates; 4 Department of Biomedical Sciences, University of Pennsylvania, Philadelphia, United States of America; 5 Pathology Department, Faculty of Medicine, Alexandria University, 21131 Alexandria, Egypt; 6 Division of Surgery and Interventional Science, University College London, London, NW3 2PS United Kingdom; 7 ASPIRE Precision Medicine Research Institute Abu Dhabi, University of Sharjah, Sharjah, United Arab Emirates; 8 BIMAI-Lab, Biomedically Informed Artificial Intelligence Laboratory, University of Sharjah, Sharjah, United Arab Emirates

**Keywords:** Renal cell carcinoma, Renal cell carcinoma, Oncogenesis

## Abstract

Gene expression is one of the most critical cellular processes. It is controlled by complex mechanisms at the genomic, epigenomic, transcriptomic, and proteomic levels. Any aberration in these mechanisms can lead to dysregulated gene expression. One recently discovered process that controls gene expression includes chemical modifications of RNA molecules by RNA-modifying proteins, a field known as epitranscriptomics. Epitranscriptomics can regulate mRNA splicing, nuclear export, stabilization, translation, or induce degradation of target RNA molecules. Dysregulation in RNA-modifying proteins has been found to contribute to many pathological conditions, such as cancer, diabetes, obesity, cardiovascular diseases, and neurological diseases, among others. This article reviews the role of epitranscriptomics in the pathogenesis and progression of renal cell carcinoma. It summarizes the molecular function of RNA-modifying proteins in the pathogenesis of renal cell carcinoma.

## Facts

- Epitranscriptomics play a central role in controlling gene expression essential to the pathogenesis of renal cell carcinoma.
- Aberrant RNA modifications have been found to contribute to the development of renal cell carcinoma and other types of cancer.
- An improved understanding of RNA modifications in renal cell carcinoma would undoubtedly contribute to developing new diagnostic and therapeutic strategies.

## Open questions

- What is the effect of aberrant RNA modifications on renal cell carcinoma progression?
- Are there any potential clinical applications of epitranscriptomics that can assist renal cell carcinoma patients?
- What are the consequences of the crosstalk between epitranscriptomics and epigenetics at the molecular level?

## Introduction

Kidney cancer had an incidence of more than 400,000 cases and more than 179,000 deaths in 2020 [1]. Around 25-30% of kidney cancer cases have metastatic disease at diagnosis time and, therefore, have a limited survival rate [2]. The most prevalent type of kidney cancer is renal cell carcinoma (RCC), accounting for 90% of kidney cancer cases [3]. RCC is a heterogeneous group of epithelial tumors with more than ten histological subtypes [4]. Clear cell RCC (ccRCC) is the most common class of RCC, accounting for 70-80%. Other common types of RCC include papillary RCC (pRCC) and chromophobe RCC (chRCC) [3]. The most commonly mutated genes in ccRCC include the von Hippel–Lindau (*VHL*) gene and chromatin-remodeling genes such as breast cancer 1 (BRCA1) associated-protein 1 (*BAP1*), SET domain-containing 2 (*SETD2*), and polybromo 1 (*PBRM1*) [5]. VHL regulates hypoxia-inducible factor (HIF) protein. Loss of VHL leads to HIF accumulation, inducing signaling pathways that lead to tumor progression [6]. *PBRM1*, *SETD2*, and *BAP1* play an essential role in chromatin remodeling and are considered co-drivers of tumor progression [7]. Bromodomain proteins (BRDs), such as bromodomain‑containing protein 9 (BRD9) and bromodomain PHD finger transcription factor (BPTF), also mediate chromatin remodeling in RCC [8].

RCC is characterized by several hallmarks, such as uncontrolled cell growth, apoptosis evasion, angiogenesis, and metabolic reprogramming. Cyclin-dependent protein kinase 2 (CDK2) contributes to impaired cell cycle regulation in RCC [9]. Tumor necrosis factor receptor-associated factor 1 (TRAF1) has antiapoptotic roles [10], and its expression was significantly decreased in RCC [11]. Integrin β4 (ITGB4) is a transmembrane protein that plays a crucial role in promoting angiogenesis [12], thus enhancing tumor invasion and metastasis. In RCC, ITGB4 was overexpressed at advanced stages [13]. Lactate Dehydrogenase A (LDHA) plays a critical role in RCC metabolism and predicts poor prognosis [14]. Other vital players in RCC metabolic reprogramming include solute carrier proteins (SLCs) [15], such as SLC1A5, which has been significantly associated with poor prognosis of ccRCC [16].

Early-stage RCC is managed through nephrectomy, while advanced stages require systemic therapies such as tyrosine kinase inhibitors (TKIs) and immune checkpoint inhibitors (ICIs) [17]. Recently, the combination of cabozantinib, a TKI, and the ICIs nivolumab and ipilimumab significantly increased the progression-free survival of advanced ccRCC patients compared with patients treated with nivolumab and ipilimumab [18]. Although combination therapy showed more effective outcomes, it may induce added toxicity compared to monotherapy [19]. Primary or acquired treatment resistances remain significant clinical challenges [20, 21]. Given these challenges, it is crucial to look for novel therapeutic targets and biomarkers of RCC.

Epitranscriptomics is the study of chemical modifications of RNA molecules occurring after RNA synthesis [22, 23]. Chemical modifications of RNA include N6-methyladenosine (m^6^A), 5-methylcytosine (m^5^C), pseudouridine (Ψ), 5-hydroxymethylcytosine (hm^5^C), and N1-methyladenosine (m^1^A) [24]. RNA is modified by several types of proteins grouped into three main categories: writers, readers, and erasers. Writer enzymes deposit the chemical modification to the RNA molecule, while erasers remove them. Reader proteins specifically detect and bind to chemically modified RNA molecules [25]. Additionally, RNA is modified by base editing, such as adenosine-to-inosine (A-to-I) and cytosine-to-uridine (C-to-U) editing [23]. Epitranscriptomic modifications were found to be involved in many diseases, including cancer [26], cardiovascular diseases [27], diabetes [28, 29], obesity [28, 30], and major depressive disorder [31].

In this review, RNA modifications related to RCC have been outlined. This review briefly discussed the mode of action of each modification and its role in cancer development with a focus on RCC. Furthermore, the crosstalk between epitranscriptomics and epigenetics, along with the modifications of noncoding RNA in the context of RCC, were reviewed.

## Aberrant RNA modifications in RCC

**Fig. 1:**
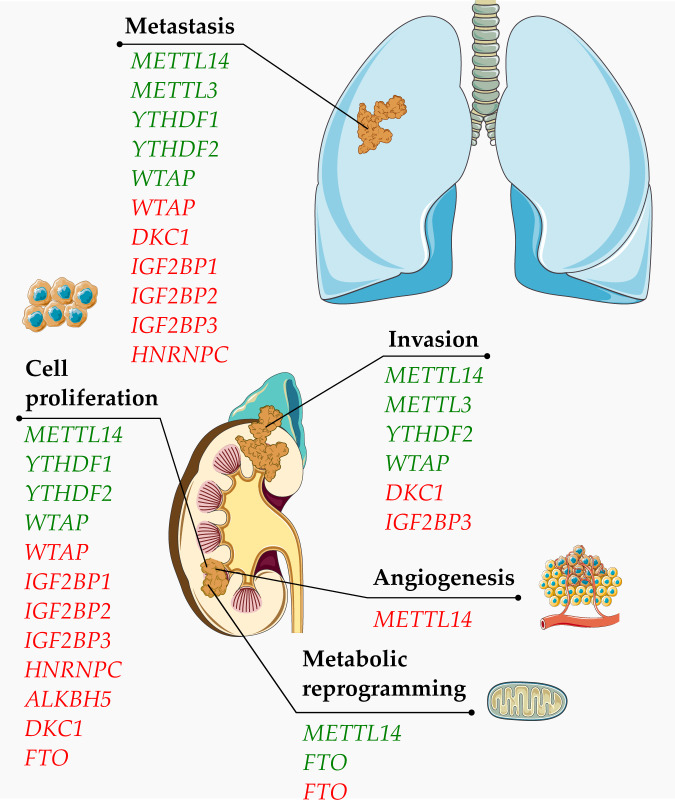
Induction of cancer hallmarks due to aberrant RNA modification in renal cell carcinoma. Green transcripts are downregulated, while red transcripts are upregulated in renal cell carcinoma.

Aberration in RNA modifications leads to RCC progression by induction of cancer hallmarks (Fig. 1). This section reviews different types of RNA modifications and their effects on cancer progression, specifically RCC. The findings of studies conducted to analyze the effects of aberrant RNA modifications on RCC are summarized in Table 1.

**Table 1:** The roles of RNA-modifying proteins and their target mRNAs in RCC.

Gene symbol	Type of RNA modification	RCC subtype	Role	Expression in RCC^a^	Target mRNA	Effect on target	Effects on cancer growth	Refs.
Writers								
*METTL14*	m^6^A	ccRCC	Tumor suppressor	↓	*PTEN*	Stabilization	Promoted migration and proliferation	[47]
*METTL14*	m^6^A	ccRCC	Tumor suppressor	↓	*ITGB4*	Suppression	Promoted metastasis	[43]
*METTL14*	m^6^A	ccRCC	Tumor suppressor	↓	*BPTF*	Suppression	Promoted glycolytic reprogramming and lung metastasis	[45]
*METTL14*	m^6^A	ccRCC	Tumor suppressor	↓	*P2RX6*	Suppression	Promoted migration and invasion	[46]
*METTL14*	m^6^A	sunitinib resistant RCC	na	↑b	*TRAF1*	Stabilization	Suppressed apoptotic and antiangiogenic effects of sunitinib	[79]
*METTL14, METTL3, WTAP*	m^6^A	NONO-TFE3 tRCC	Tumor suppressor	↓	*PARP1*	Suppression	Promoted cell proliferation, migration, and invasion	[61]
*WTAP*	m^6^A	ccRCC, pRCC	Oncogene	↑	*S1PR3*	Stabilization	Promoted migration and proliferation	[53]
*WTAP*	na	RCC	Oncogene	↑	*CDK2*	Stabilization	Promoted proliferation	[52]
*NSUN1 (NOP2)*	m^5^C	ccRCC	na	↑	na	na	na	[110, 111]
*NSUN2*	m^5^C	ccRCC	na	↑	na	na	na	[110]
*NSUN4*	m^5^C	ccRCC	na	↓	na	na	na	[110, 111]
*NSUN5*	m^5^C	ccRCC	Oncogene	↑	*ENO3*	Stabilization	Accompanied by increased tumor size, involved in Warburg effect and tumor progression	[112]
*NSUN5*	m^5^C	ccRCC	na	↑	na	na	na	[110]
*NSUN6*	m^5^C	ccRCC	na	↑	na	na	na	[111]
*DKC1*	Ψ	ccRCC	Oncogene	↑	na	na	Promoted cell proliferation, migration, and invasion	[120]
Readers								
*YTHDF1*	m^6^A	ccRCC	Tumor suppressor	na	*PTEN*	Stabilization	Promoted migration and proliferation	[47]
*YTHDF2*	m^6^A	ccRCC	Tumor suppressor	↓	*ITGB4*	Suppression	Promoted metastasis	[43]
*YTHDF2*	m^6^A	NONO-TFE3 tRCC	Tumor suppressor	↓	*PARP1*	Suppression	Promoted cell proliferation, migration, and invasion	[61]
*IGF2BP1*	m^6^A	ccRCC	Oncogene	↑	*LDHA*	Stabilization	Facilitated aerobic glycolysis and accelerated energy metabolism	[77]
*IGF2BP1, IGF2BP2, IGF2BP3*	m^6^A	ccRCC, pRCC	Oncogene	↑	*S1PR3*	Stabilization	Promoted migration and proliferation	[53]
*IGF2BP3*	m^6^A	ccRCC	Oncogene	↑	*CDK4, COL6A1, LAMA5, FN1*	Stabilization	Promoted cell proliferation, migration, and invasion	[137]
*HNRNPC*	m^6^A	pRCC	Oncogene	↑	na	na	Promoted cell proliferation and migration	[82]
Erasers								
*ALKBH1*	m^6^A	RCC	Oncogene	↑	*GPR137*	Upregulation	Promoted cell viability and migration	[95]
*ALKBH3*	m^6^A	RCC	Oncogene	↑	na	na	na	[94]
*ALKBH5*	m^6^A	RCC	Oncogene	↑	*AURKB*	Stabilization	Promoted cell proliferation	[92]
*ALKBH5*	m^6^A	ccRCC	Tumor suppressor	↓	na	na	na	[91]
*ALKBH5*	m^6^A	RCC	Oncogene	↑	na	na	Promoted cell migration and proliferation by modulating cell cycle and epithelial-mesenchymal transition	[93]
*FTO*	m^6^A	ccRCC	Tumor suppressor	↓	*PGC-1α*	Stabilization	Increased mitochondrial biogenesis and oxidative stress	[101]
*FTO*	m^6^A	VHL deficient ccRCC	Oncogene	↑	*SLC1A5*	Stabilization	Metabolic reprogramming, cell survival	[104]
*FTO*	m^6^A	HIF2α^low/−^ ccRCC	Oncogene	↑	*BRD9*	Stabilization	Facilitated tumor growth	[105]
*FTO*	m^6^A	RCC	Oncogene	↑	na	na	Promoted cell migration and proliferation by modulating cell cycle and epithelial-mesenchymal transition	[93]
*TET2*	m^5^C	ccRCC	na	↓	na	na	na	[110, 111]
Pseudouridine								
*DKC1*	U-to-Ψ	ccRCC	Oncogene	↑	na	na	Promoted cell proliferation, migration, and invasion	[120]
Base editing								
*APOBEC3G*	C-to-U	ccRCC	na	↑	na	na	na	[132]

*Na* not available.

^a^Expression level compared to normal control, unless otherwise specified. ↑: upregulated, ↓: downregulated.

^b^Compared to sunitinib-sensitive RCC cells.

### N6-methyladenosine

**Fig. 2:**
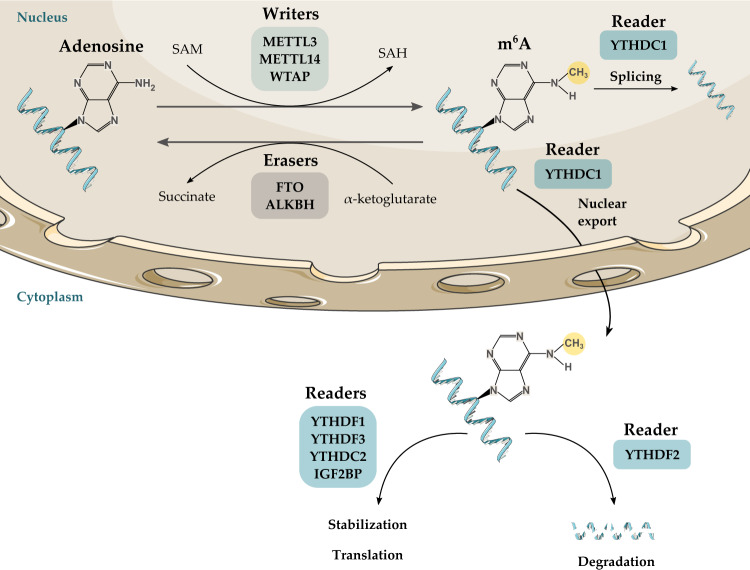
Writers, readers, and erasers of N6-methyladenosine. Modification of adenosine to N6-methyladenosine is a dynamic process mediated by the writer enzymes, including METTL3, METTL14, and WTAP. Methylation is catalyzed by SAM as a cofactor and methyl group donor. FTO and ALKBH reverse the process along with the conversion of α-ketoglutarate to succinate. YTHDC1 reads N6-methyladenosine and mediates mRNA splicing and nuclear export. In the cytoplasm, YTHDF1, YTHDF3, and YTHDC2 stabilize the mRNA molecule and mediate its translation, while YTHDF2 mediates mRNA degradation. M^6^A N6-methyladenosine, METTL methyltransferase-like protein, WTAP Wilms’ tumor 1-associating protein, SAM S-adenosylmethionine, SAH S-adenosylhomocysteine, FTO fat mass and obesity-associated protein, ALKBH ALKB homolog, YTHDC YTH domain-containing protein, YTHDF YTH domain-family protein, IGF2BP Insulin-like growth factor 2 binding protein.

Methylation of RNA at the N6 position of adenosine resulting in m^6^A is the most characterized and abundant RNA methylation. It is a dynamic modification that involves several proteins acting as writers, readers, and erasers of m^6^A [26] (Fig. 2). This section will discuss recent findings about proteins involved in m^6^A deposition, detection, and removal and their roles in RCC progression.

#### Writers

M^6^A methyltransferases are proteins that modify RNA with m^6^A. This reaction is catalyzed by multicomponent methyltransferase complexes. Most of the m^6^A modifications of RNA molecules occur by methyltransferase like 3 (METTL3), METTL14, and Wilms’ tumor 1-associating protein (WTAP) complex that catalyzes methylation of internal adenosine residues to form m^6^A [32]. The methylation reaction catalyzed by this complex needs S-adenosylmethionine (SAM) as a cofactor and methyl-group donor. Upon adenosine methylation, SAM is converted to S-adenosylhomocysteine (SAH). METTL3 was identified as the catalytic subunit of this complex, catalyzing the methylation reaction and crosslinking with SAM. The substrate RNA is recognized by the METTL14 molecule, which also plays a role in stabilizing the complex. WTAP provides a scaffolding function and localizes the complex at the nuclear speckles [33, 34]. M^6^A deposition is guided by histone H3 trimethylation at lysine 36 (H3K36me3). METTL14 binds to H3K36me3 and deposits m^6^A co-transcriptionally, indicating complex crosstalk between histone modifications and RNA methylation [35].

Depending on the type of cancer, the m^6^A methyltransferase complex can either suppress or promote tumor growth. For example, METTL3 and METTL14 exhibited an oncogenic role in acute myeloid leukemia (AML), in which they were highly expressed and correlated with shorter survival [36]. Additionally, WTAP was found to play an oncogenic role in AML [37]. In contrast, METTL3 and METTL14 showed a tumor-suppressive role in glioblastoma stem cells [38]. The functions of the components within the m^6^A writing complex are connected. For example, METTL3 was found to regulate WTAP homeostasis [39]. Interestingly, although WTAP forms a complex with METTL3, many unique target RNAs are associated with only one of the proteins in the complex [34]. Other methyltransferases play a role in m^6^A methylation. METTL16 methylates mRNAs involved in the SAM synthesis [40]. METTL7B has a tumorigenesis role by inducing cancer cell proliferation in non-small cell lung carcinoma (NSCLC) [41].

METTL14 showed a tumor-suppressive effect in RCC and was downregulated compared to normal controls. Higher expression of METTL14 was associated with better survival [42]. Upon METTL14 downregulation in ccRCC, *ITGB4* was overexpressed and promoted invasiveness and metastasis [43]. METTL14 was found to suppress the NUF2 component of NDC80 kinetochore complex (*NUF2*), cell division cycle associated 3 (*CDCA3*), and kinesin family member 14 (*KIF14*), leading to ccRCC progression upon downregulation of METTL14 [44]. Similarly, downregulated METTL14 induced an accumulation of BPTF [45] and P2X purinoceptor 6 (P2RX6), an ATP receptor [46], driving metastasis and invasion in ccRCC. Therefore, methylation induced by METTL14 suppressed *BPTF* and *P2RX6* expression. In contrast to the previous studies, METTL14 stabilized *PTEN*, leading to tumor progression inhibition and suggesting a potential therapeutic target [47].

METTL7B was found to be upregulated in ccRCC compared to normal controls. Its expression was significantly associated with tumor size, lymph node metastasis, and poor prognosis [48]. In contrast, METTL7A expression was considerably lower in renal cancer than in normal tissues, and its low expression was associated with poor prognosis [49]. METTL3 played a tumor-suppressive role in the RCC, and its downregulation was associated with larger tumor size, higher histological grade, and poor survival [50, 51]. In RCC, WTAP was found to maintain the stability of *CDK2* mRNA, which in turn promotes the proliferation of RCC cells [52]. Additionally, WTAP mediated m^6^A modification of sphingosine-1-phosphate receptor 3 (*S1PR3*). S1PR3 was found to induce RCC proliferation and metastasis by activating the phosphatidylinositol 3‑kinase (PI3K)/protein kinase B (AKT) pathway [53].

#### Readers

Reader proteins detect m^6^A and create a biological signal. Mainly, these proteins belong to 4 families: YT521-B homology (YTH) domain-containing proteins, insulin-like growth factor 2 binding protein (IGF2BP) family, heterogeneous nuclear ribonucleoprotein (hnRNP), and proline-rich and coiled-coil-containing protein 2 A (PRRC2A) [26]. Most of the studies that investigated RCC were conducted on YTH domain-containing proteins, IGF2BP, and hnRNP, which will be the focus of this section.

##### YTH domain-containing proteins

The YTH domain-containing proteins include YTH domain family proteins (YTHDFs) and YTH domain-containing proteins (YTHDCs). In eukaryotes, these proteins are expressed by five genes: *YTHDF1*, *YTHDF2*, *YTHDF3*, *YTHDC1*, and *YTHDC2*. After recognizing m^6^A modification of target genes, YTH domain-containing proteins recruit different complexes to regulate several processes, such as RNA translation, RNA decay, RNA splicing, and nuclear export. Furthermore, they play various roles in cancer development and progression [54].

YTHDF2 was the first characterized member of YTH domain-containing proteins. It was found to promote target decay. YTHDF2 was upregulated in lung adenocarcinoma and was found to degrade axis inhibition protein 1 (*AXIN1*), which encodes a negative regulator of the Wnt/β-catenin signaling, eventually leading to Wnt/β-catenin activation and tumor progression [55]. In contrast, YTHDF1 and YTHDF3 were found to stabilize their targets and promote translation. Upregulation of YTHDF1 was found to be associated with poor prognosis of ovarian cancer. Mechanistically, YTHDF1 was found to augment the translation of eukaryotic translation initiation factor 3 subunit C (*EIF3C*) in an m^6^A-dependent manner. Thus promoting the overall translation output, which in turn initiates ovarian cancer tumorigenesis and metastasis [56]. Moreover, YTHDF3 overexpression was clinically correlated with brain metastasis of breast cancer by promoting the translation of crucial brain metastatic genes ST6 N-acetylgalactosaminide alpha-2,6-sialyltransferase 5 (*ST6GALNAC5*) and gap junction protein alpha 1 (*GJA1*) [57]. YTHDC1 was found to play roles in mRNA splicing and nuclear export, while YTHDC2 was found to promote translation [54]. In triple-negative breast cancer, YTHDC1 had an oncogenic role by promoting *SMAD3* mRNA nuclear export and expression to augment the transforming growth factor-β (TGF-β) signaling cascade [58]. YTHDC2 promoted gastric cancer progression via increasing the translation of yes-associated protein (*YAP*) oncogene [59].

In ccRCC, the expression of YTHDF1-3 and YTHDC1 were significantly down-regulated compared to the normal tissue [60]. YTHDF1 played a role in stabilizing m^6^A-modified *PTEN* and was found to suppress tumor progression in ccRCC by inhibiting the activation of the PI3K/AKT signaling pathway [47]. Moreover, YTHDF2 was found to be downregulated in ccRCC and acted by promoting the decay of *ITGB4* mRNA [43]. Additionally, in NONO-TFE3 translocation renal cell carcinoma (NONO-TFE3 tRCC), a subtype of RCC associated with Xp11.2 translocation/TFE3 gene fusions RCC (Xp11.2 tRCCs), YTHDF2 played a tumor-suppressive role by suppressing poly(ADP-ribose) polymerase 1 (*PARP1*) expression. The downregulation of YTHDF2 promoted cell proliferation, invasion, and migration [61].

##### Insulin-like growth factor 2 binding proteins

IGF2BPs, also known as insulin mRNA binding proteins (IMPs), are another group of m^6^A readers, consisting of IGF2BP1, IGF2BP2, and IGF2BP3. IGF2BPs are expressed in most embryonic tissues and play crucial roles in embryogenesis by controlling RNA localization, stability, and translation [62, 63]. IGF2BPs recognize m^6^A-modified RNAs through their K homology (KH) domain and function by stabilizing their target RNA [64]. In adults, IGF2BP2 is widely expressed in different tissues. In contrast, the expression levels of IGF2BP1 and IGF2BP3 are negligible in adults except in reproductive organs [65]. Therefore, they are considered oncofetal proteins since they are severely upregulated in various tumors.

IGF2BPs were found to be overexpressed and play oncogenic roles in different tumor types. They promote cancer progression by stabilizing methylated mRNAs of oncogenic targets [66]. In breast cancer, IGF2BP1 was found to maintain the stability of m^6^A-modified *c-Myc* mRNA in vivo [67]. IGF2BP2 and IGF2BP3 enhanced the stability of methylated ephrin type-A receptor 2 (*EPHA2*) and vascular endothelial growth factor A (*VEGFA*) mRNAs, respectively, in colorectal cancer cells. EPHA2 and VEGFA activate both PI3K/AKT and the extracellular signal-regulated kinase 1/2 (ERK1/2) signaling pathways to induce tumor progression by increasing tumor cell proliferation and vasculogenic mimicry [68]. Vasculogenic mimicry occurs when tumor cells form microvascular channels that provide blood supply in aggressive tumors independently of tumor angiogenic mechanisms [69].

Overexpression of IGF2BP3 in ccRCC was associated with advanced stage and grade of primary tumors, coagulative tumor necrosis, and sarcomatoid differentiation [70]. *IGF2BP3* was significantly overexpressed in metastatic RCC and primary RCC that were likely to develop metastasis. It was suggested as a diagnostic marker to identify patients with a high risk of developing metastasis [70–74]. Similarly, patients with localized pRCC and chRCC overexpressing *IGF2BP3* were over ten times more likely to develop metastasis than patients with low *IGF2BP3* expression [75]. Moreover, high levels of circulating IGF2BP3 were detected in RCC patients with high-grade and more aggressive tumors and were independently associated with poor survival. Hence, it suggests its potential application as a minimally invasive sampling method that can improve therapeutic planning [76].

In RCC, the expression of IGF2BPs could be increased by early growth response 2 (EGR2) transcription factor. IGF2BPs, in turn, enhance the stability of the *S1PR3* mRNA [53]. IGF2BP1 was upregulated in ccRCC cell lines and facilitated tumor energy metabolism by promoting glycolysis [77]. Moreover, the upregulation of IGF2BP3 activated the nuclear factor kappa B (NF-кB) pathway, which promoted RCC cell migration and invasion [78]. Furthermore, IGF2BP2 was involved in stabilizing *TRAF1* by m^6^A RNA methylation via METTL14 in sunitinib-resistant RCC cells. Ultimately, this led to suppressing the apoptotic and antiangiogenic effects of sunitinib [79]. Overall, these studies suggest the oncogenic role of IGF2BPs in RCC, and due to their oncofetal transcription, they are considered a potential therapeutic target.

##### Heterogeneous nuclear ribonucleoproteins

Another group of m^6^A reader proteins includes hnRNPs, which are responsible for pre-mRNA maturation into functional mRNA, mRNA stabilization, and translocation. Up to date, twenty members of the hnRNP family have been identified and termed hnRNP A-U [80]. HnRNPs were found to be involved in apoptosis, epithelial-mesenchymal transition (EMT), and cancer angiogenesis [81]. HnRNPC was upregulated in pRCC, promoting cell proliferation and migration in vitro [82]. More extensive studies are needed to investigate the role of hnRNP proteins in RCC pathogenesis.

#### Erasers

M^6^A erasers remove methyl groups from RNA bases. This group of RNA-modifying proteins includes members of the AlkB homolog (ALKBH) family, which consists of ALKBH 1-8 and fat mass and obesity-associated protein (FTO). They catalyze the demethylation of nucleic acid bases by oxidation reaction dependent on iron (II) and α-ketoglutarate [83]. The demethylation reaction converts α-ketoglutarate to succinate, which is accompanied by the release of formaldehyde and carbon dioxide [84].

The expression level of ALKBH genes varies in different cancers. A tumor-suppressive effect of ALKBH5 was detected in pancreatic cancer by preventing its progression by activating period circadian regulator 1 (*PER1*), which in turn inhibited cell growth [85]. Moreover, ALKBH5 inhibited the metastasis of colon cancer [86]. On the contrary, ALKBH5 promoted invasion and metastasis in gastric cancer [87]. In addition, it inhibited autophagy of epithelial ovarian cancer by enhancing the stability of BCL2 apoptosis regulator (*BCL2*), which has an antiapoptotic role [88]. Additionally, ALKBH5 promoted NSCLC progression by repressing tissue inhibitors of metalloproteinase 3 (*TIMP-3*) [89].

ALKBH5 was differentially expressed between RCC subtypes and oncocytomas, suggesting its potential application as a diagnostic marker [90]. Studies have shown inconsistent results regarding its expression in RCC and adjacent normal tissue. Some studies suggested that the expression of ALKBH5 was significantly downregulated in ccRCC compared to normal tissue. Additionally, it was correlated with shortened overall and cancer-specific survival [91]. On the other hand, other studies suggested an oncogenic role of ALKBH5, which was overexpressed in RCC. Moreover, elevated expression of ALKBH5 was correlated with larger tumor volume, higher TNM staging, and worse prognosis. Mechanistically, ALKBH5 expression was upregulated by HIF induced by hypoxia. ALKBH5 stabilized aurora kinase B (*AURKB)*, promoting RCC cell proliferation [92]. Additionally, ALKBH5 was found to promote cell proliferation and migration through cell cycle alteration and EMT [93]. A similar oncogenic effect was detected in ALKBH3 in RCC patients, in which its expression was positively correlated with advanced TNM staging and poor prognosis [94]. Moreover, ALKBH1 had an oncogenic role in RCC cell lines and promoted cell migration and viability [95]. Overall, further studies are required to elucidate the molecular mechanisms behind the different roles of ALKBH in RCC.

Like ALKBH, FTO also showed conflicting effects, with studies reporting tumor-suppressive effects while others reported oncogenic effects. Jeschke et al. found that FTO presented a tumor-suppressive role in many epithelial tumors, including breast, prostate, cervical, liver, and lung cancers. This was shown, upon FTO depletion, by the induction of Wnt signaling and EMT transition [96]. Similarly, FTO exhibited a suppressive effect on prostate cancer proliferation and metastasis by stabilizing chloride intracellular channel 4 (*CLIC4*), which encodes a protein that can inhibit cell proliferation through the TGF-β pathway [97].

On the contrary, a recent study utilized epitranscriptomic landscape mapping and revealed that positive FTO expression was associated with poor survival of breast cancer [98]. FTO was elevated in gastric cancer and showed an oncogenic effect by promoting cell proliferation and metastasis via inducing the degradation of caveolin-1 (*CAV1*) mRNA by demethylation. As a result of *CAV1* degradation, mitochondrial dynamics are altered, leading to an elevated ATP level and thereby favoring cancer growth [99]. Another example of the oncogenic role of FTO was observed in pancreatic cancer, where FTO induced cancer progression via stabilizing the mRNA of platelet-derived growth factor C (*PDGFC*). Eventually, this led to the reactivation of the PI3K/AKT signaling pathway that promoted cell growth [100].

The dichotomous effect of FTO was also observed in RCC. Decreased FTO level correlated with increased tumor severity and poor overall and cancer-specific survival following nephrectomy, suggesting a tumor-suppressive role of FTO [91, 101]. Moreover, in VHL-deficient ccRCC cells with ectopic FTO expression, the expression of peroxisome proliferator-activated receptor γ coactivator 1 α (*PGC-1α*) increased due to decreased m^6^A levels in its transcript. PGC-1α restored mitochondrial activity, which was revealed by an elevated ATP level and induced oxidative stress and ROS production. Consequently, this resulted in impaired tumor growth [101]. This finding contradicts what was found in gastric cancer, where the ATP reduction induced by FTO depletion restricted cancer growth [99]. This could be due to the opposing effects of ATP within the tumor microenvironment, where it can promote or inhibit cancer growth depending on its concentration, receptors expressed by cancer and immune cells, and the expression of ectonucleotidase enzymes that hydrolyze ATP [102, 103].

Besides its tumor-suppressive effect in RCC, FTO also exhibited an oncogenic effect. It modulated EMT and cell cycle, promoting cell proliferation and migration in RCC cell lines [93]. A study conducted by Xiao et al. found that FTO was overexpressed in ccRCC tumors with *VHL* deletions or mutations compared to adjacent normal tissue. Additionally, they detected a synthetic lethal interaction between FTO and VHL. FTO inhibition selectively reduced the growth of VHL-deficient cells in vitro and in vivo in a HIF-independent manner. Furthermore, they identified the glutamine transporter SLC1A5 as a target of FTO, leading to the metabolic reprogramming and survival of *VHL*-deficient cells [104]. Moreover, FTO showed an oncogenic role in HIF2α^low/−^ ccRCC by stabilizing the mRNA of *BRD9*. Inhibition of *BRD9* in BALB/c mice bearing HIF2α^low/−^ ccRCC cell line–derived xenografts and patient-derived tumor xenografts led to tumor growth inhibition and prolonged survival with greater efficacy than sunitinib [105]. This indicates that cells with different genetic backgrounds respond to FTO differently. The context-dependent role of FTO as oncogenic or tumor-suppressive is complex and requires further investigation of its underlying molecular mechanisms.

### 5-Methylcytosine (m^5^C)

**Fig. 3:**
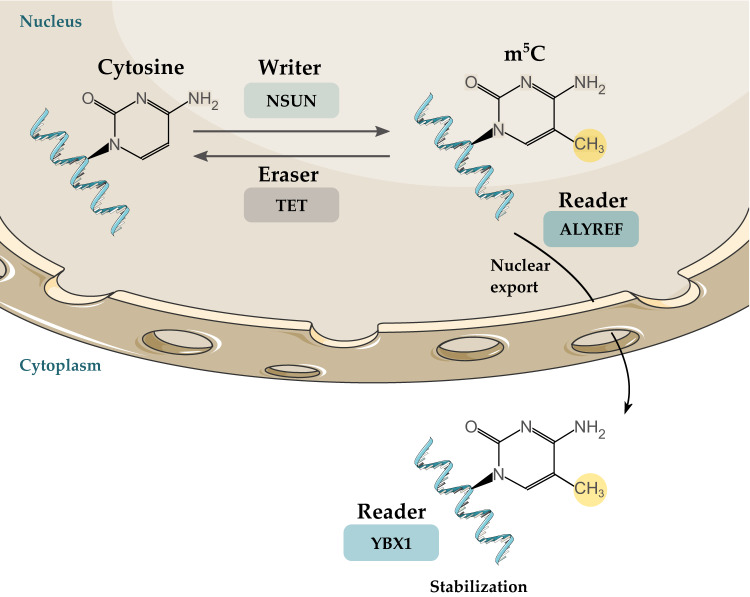
Writers, readers, and erasers of 5-methylcytosine. Modification of cytosine to 5-methylcytosine is mediated by NSUN proteins. This process is reversed by TET. ALYREF is a reader protein that mediates the nuclear export of mRNA, while YBX1 regulates mRNA stability in the cytoplasm. M^5^C 5-methylcytosine, NSUN NOL1/NOP2/SUN domain family member, TET ten-eleven translocation, ALYREF Aly/REF export factor, YBX1 Y-box binding protein 1.

Methylation at position 5 of cytidine residue can occur in mRNA, rRNA, tRNA, and other noncoding RNA molecules [26]. Similar to m^6^A modifications, there is a group of m^5^C writers, readers, and erasers. M^5^C writers include seven members of the NOL1/NOP2/SUN domain family member (NSUN) family, NSUN1 to NSUN7, and DNA methyltransferase-2 (DNMT2). Reader proteins that bind to m^5^C include YTHDF2, Aly/REF export factor (ALYREF), and Y-box binding protein 1 (YBX1) [106]. ALYREF mediates the nuclear export of mRNA, while YBX1 regulates mRNA stability in the cytoplasm [107]. M^5^C demethylation occurs via the eraser molecule ten-eleven translocation (TET) [106] (Fig. 3).

M^5^C was found to alter the progression of different types of cancers. In breast cancer, the overexpression of *NSUN2* was correlated with increased metastasis and invasion [108]. *ALYREF* and *NSUN5* were overexpressed at the metastasis stage of head and neck squamous cell carcinoma [109]. In ccRCC, *NSUN1*, *NSUN2*, *NSUN5*, and *NSUN6* were upregulated, while *NSUN4* and *TET2* were downregulated [110, 111]. NSUN5 promoted ccRCC progression through the Warburg effect and increased cell growth through stabilizing enolase 3 (*ENO3*) mRNA [112]. Warburg effect is the phenomenon of cells undergoing anaerobic glycolysis for energy production even under normal oxygen concentration [113]. Another study on ccRCC revealed the role of YBX1 in stabilizing phosphatidylethanolamine binding protein 1 (*PEBP1*). YBX1 is recruited to m^5^C-containing *PEBP1* mRNA by phosphatidylethanolamine binding protein 1 pseudogene 2 (*PEBP1P2*). The low expression of *PEBP1P2* is correlated with poor prognosis and advanced stages of the ccRCC [114]. Further studies are required to elaborate on the molecular mechanism of m^5^C regulators and how they affect RCC progression.

### Pseudouridine

**Fig. 4:**
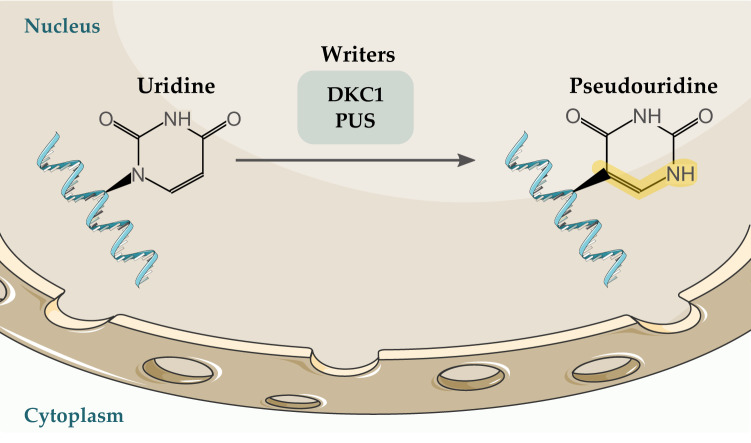
Modification of uridine to pseudouridine is mediated by DKC1 and PUS proteins. DKC1 dyskerin pseudouridine synthase 1, PUS pseudouridine synthase.

Uridine can be converted to pseudouridine (Ψ), a C5-glycoside isomer of uridine, by Ψ synthase. Ψ was initially detected in rRNA and tRNA. Later, it was also detected in mRNA, long noncoding RNA (lncRNA), and small nuclear RNA (snRNA) molecules [115]. It is involved in several physiological roles depending on the modified RNA molecule. It influences rRNA folding and ribosome assembly in rRNA and alters tRNA interaction with rRNA and mRNA [116]. In mRNA, Ψ affects stability, promotes pre-mRNA splicing, and mediates translation [117]. In eukaryotes, dyskerin pseudouridine synthase 1 (DKC1) and several proteins belonging to the pseudouridine synthase (PUS) family function as Ψ writers (Fig. 4) [26]. In breast cancer, *DKC1* overexpression predicted poor prognosis [118]. Furthermore, *PUS7* was upregulated in ovarian cancer and has been suggested as a potential biomarker [119]. In ccRCC, DKC1 was found to have oncogenic roles by promoting cell proliferation, migration, and invasion via the NF-κB pathway [120].

### RNA editing

**Fig. 5:**
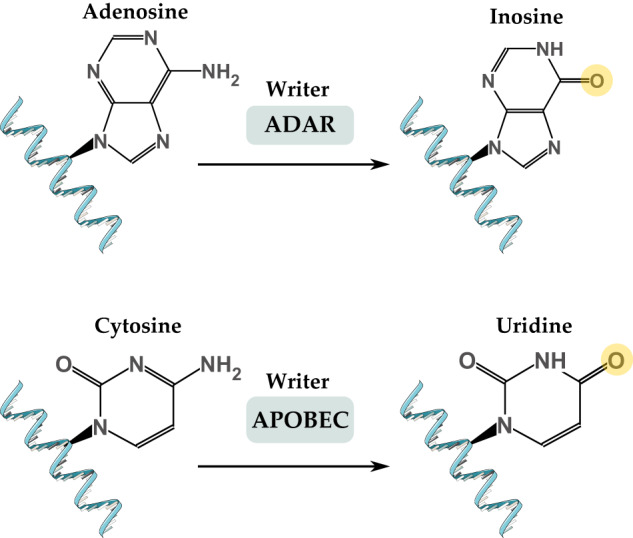
RNA editing involves deamination of adenosine to inosine mediated by ADAR proteins and cytosine to uridine mediated by APOBEC proteins. ADAR adenosine deaminase acting on dsRNA, APOBEC apolipoprotein B mRNA-editing cytosine deaminase.

In addition to the chemical modifications of RNA bases that do not affect RNA sequence, RNA bases can be modified by deamination, resulting in the conversion of the RNA base type. There are two types of RNA editing: A-to-I editing and C-to-U editing (Fig. 5) [23].

#### Adenosine to inosine editing

A-to-I editing is the most prevalent RNA editing in vertebrates. It is mediated by adenosine deaminase acting on dsRNA 1 (ADAR1) and ADAR2. ADARs were found to be associated with splicing factors, indicating their potential roles in alternative splicing and transcriptional control [121]. Additionally, ADARs target RNA viruses by inducing A-to-I hypermutations. They have both pro- and anti-viral effects [122]. Moreover, ADARs were found to play roles in cancer progression. In breast cancer, upregulation of ADAR1 promoted cancer progression through cell cycle regulation and controlling DNA damage response [123]. Based on data from the Cancer Genome Atlas (TCGA), there was no significant difference in ADAR1 expression in ccRCC compared to normal renal tissue, and it did not have an impact on patients’ survival [124].

#### Cytosine to uridine editing

Apolipoprotein B mRNA-editing cytosine deaminase (APOBEC) catalyzes cytosine deamination mediating C-to-U conversion. APOBEC family includes activation-induced cytidine deaminase (AID), APOBEC1 (A1), APOBEC2 (A2), APOBEC3 (A3), and APOBEC4 (A4). A3 comprises seven members– A3A, A3B, A3C, A3D, A3F, A3G, A3H [125]. Members of the APOBEC family play various physiological roles, and all of them have C-to-U deaminase activity except A2 and A4 [126]. A1, A3A, and A3G were found to act on single-stranded RNA as a substrate [127]. A1 regulates lipid metabolism by mediating dietary lipid uptake from the intestine [128]. A3 deaminates viral DNA or RNA, leading to viral restriction, which is the degradation of the viral genome when the mutational load is so high that the genome cannot function properly [127, 129]. Deamination of A3 can support viral evolution provided it does not lead to viral restriction and the resulting mutations are fixed in the viral genome [127].

Besides its role in viral restriction and evolution, APOBEC has a similar effect on cancer progression, where it can lead to tumor restriction or tumor evolution. Deamination induced by APOBEC initiates mutagenesis in cancer cells, leading to autonomous lethality and tumor restriction. Mutations introduced by APOBEC can lead to chromosomal instability. Pecori et al. hypothesized that the inflammatory microenvironment of cancer leads to elevated expression of A3 that induces localized hypermutation attempting to kill malignant cells [127].

APOBEC proteins were also involved in generating tumor heterogeneity when the level of the introduced mutations was low. This will eventually lead to tumor progression [127]. For example, inhibition of lncRNA H19 and its target A3G due to sulforaphane treatment inhibited pancreatic cancer progression in vitro and in vivo by inhibiting TGF-β-induced SMAD2 phosphorylation [130]. This indicates that despite the mutation surge induced by APOBEC that promotes the evolution of more aggressive clones, it also provides potential targets for cytotoxic and immunotherapies [131]. Furthermore, A3G was found to be an unfavorable prognostic marker in ccRCC patients. The expression of A3G was positively correlated with the expression of several immunoinhibitors and the presence of immunosuppressive cells [132].

## The crosstalk between epigenetics and RNA modifications

Recently, many studies have shown an integration between epitranscriptomic and epigenetic modifications, such as histone modifications [133] and DNA methylation [134]. This integration has been observed in many physiological and pathogenic processes and can influence chromatin accessibility and transcription regulation. METTL3 has been found to play a role in mammalian development by regulating the heterochromatin of mouse embryonic stem cells [135]. In glioblastoma, METTL3 modified and promoted the expression of genes involved in histone modifications in an m^6^A-dependent manner [136]. Moreover, METTL3 was found to facilitate the demethylation of nearby genomic DNA in an m^6^A-dependent manner in cancer and normal cells. Upon RNA methylation by METTL3, fragile-X mental retardation autosomal 1 (FXR1) protein recognizes m^6^A and recruits TET1 protein to demethylate DNA in a process called RNA methylation-coupled DNA demethylation [134].

An example of the crosstalk between epigenetic and epitranscriptomic regulations in ccRCC is the interaction between IGF2BP3 and a lncRNA cofactor called DNA methylation–deregulated and RNA m^6^A reader–cooperating lncRNA (*DMDRMR*). DNA hypomethylation of its promoter region induces DMDRMR expression. IGF2BP3 and DMDRMR stabilized target mRNAs, such as cyclin-dependent kinase 4 (*CDK4*) and three extracellular matrix components: collagen type VI alpha 1 chain (*COL6A1*), laminin subunit alpha 5 (*LAMA5*), and fibronectin 1 (*FN1*). Consequently, activation of CDK4 led to accelerating ccRCC cell proliferation, and FN1 partially promoted invasion and metastasis. Moreover, the elevated expression of *IGF2BP3* and *DMDRMR* was associated with poor overall survival [137].

Another component of the epigenetic machinery includes noncoding RNA molecules. Initially, scientists thought that RNA modifications occur only in mRNA molecules. With the advance in detection technologies, recent studies detected modifications in noncoding RNA, such as rRNAs, tRNAs, microRNAs (miRNAs), lncRNAs, and small nucleolar RNAs [32]. Additionally, noncoding RNAs can regulate the expression of RNA-modifying proteins. In RCC, several studies detected noncoding RNAs as regulators or targets of RNA-modifying proteins, eventually controlling the expression of downstream genes involved in tumorigenesis (Table 2).

**Table 2:** Noncoding RNAs and RNA modifications in RCC.

RNA-modifying protein	Protein’s inhibitor	Protein’s target	Involvement in RCC	Ref
WTAP	miR-501-3p	na	Downregulation of miR-501-3p promotes disease progression	[138]
IGF2BP1	miR-372	na	Downregulation of miR-372 promotes cell proliferation and invasion	[139]
FTO	miR-155	na	miR-155 enhances cell proliferation	[140]
IGF2BP3	na	*CDKN2B-AS1*	IGF2BP3 stabilizes *CDKN2B-AS1* promoting cell growth and metastasis	[141]
METTL-14	na	*lnc-LSG1*	Downregulation of METTL-14 leads to *lnc-LSG1* binding to and degrading its target ESRP2 leading to metastasis.	[142]
METTL-14	na	*NEAT1_1*	Downregulation of METTL-14 leads to *NEAT1_1* stabilization which promotes a malignant phenotype	[143]

Noncoding RNA can inhibit or be targeted by RNA-modifying proteins. *Na* not available.

### Noncoding RNAs as regulators

*WTAP* is targeted and silenced by miR-501-3p, which was found to be downregulated in RCC. The overexpression of miR-501-3p inhibited disease progression [138]. Another inhibitory effect of miRNA was found against *IGF2BP1*, which was inhibited by miR-372 in RCC by direct interaction with its putative binding site at 3’-UTR. In RCC cell lines and tissue samples, miRNA-372 was down-regulated, and when miRNA-372 was overexpressed, it inhibited RCC cell proliferation and invasion. This suggests that miRNA-372 has therapeutic potential in the treatment of RCC [139]. Moreover, ccRCC cell lines that were treated with miR-155 exhibited an inhibition of *FTO*, resulting in increased tumor cell proliferation [140].

The lncRNA TRAF3IP2 antisense RNA 1 (*TRAF3IP2-AS1*) functions by binding to *PARP1* mRNA and recruiting m^6^A methyltransferase complex consisting of METTL3, METTL14, and WTAP, leading to *PARP1* degradation. In NONO-TFE3 tRCC, *TRAF3IP2-AS1* was downregulated, which resulted in PARP1 accumulation and promoted tumorigenesis [61].

### Noncoding RNAs as targets

In ccRCC, IGF2BP3 was found to stabilize a lncRNA called cyclin-dependent kinase inhibitor 2B antisense 1 (*CDKN2B-AS1*). *CDKN2B-AS1* was significantly upregulated in ccRCC and participated in epigenetic activation of *NUF2*. Thus, it enhanced *NUF2* transcription, which promoted tumor cell growth and metastasis both in vitro and in vivo. Patients with elevated *IGF2BP3*, *CDKN2B-AS1*, and *NUF2* showed reduced survival time [141].

Furthermore, epithelial splicing regulatory protein 2 (ESRP2) inhibits metastasis and is ubiquitinated by *lnc-LSG1*, causing ESRP2 protein degradation. METTL14 was found to target *lnc-LSG1* by m^6^A modification, which inhibits its binding to ESRP2 via YTHDC1. As a result, ESRP2 will be protected from degradation and inhibit metastasis. In ccRCC, METTL14 is downregulated, leading to the binding of ESRP2 to *lnc-LSG1* and ESRP2 degradation [142].

Moreover, METTL14 was found to downregulate the expression of the oncogenic long noncoding RNA nuclear enriched abundant transcript 1_1 (*NEAT1_1*) in an m^6^A-dependent manner. YTHDF2 detected m^6^A modification on *NEAT1_1*, leading to *NEAT1_1* degradation. Downregulation of METTL14 in ccRCC will lead to the stabilization of the *NEAT1_1*, resulting in a malignant phenotype [143].

## Conclusion and future perspectives

Several decades following the discovery of RNA modifications, the role of RNA modifications in cancer was not fully understood [144]. Recently, the advancement of detection methods of RNA modifications led to a dramatic burst in identifying their functional roles in health and disease. The varying functions of some RNA-modifying proteins, which can either promote or inhibit RCC progression, provide a better understanding of the heterogenous RCC environment. The aberrant expression of RNA-modifying proteins has been found to induce cancer hallmarks in RCC, such as cell proliferation, metabolic programming, angiogenesis, invasion, and metastasis. Moreover, RNA modifications play an essential role in developing several physiological conditions that can increase the risk of RCC, such as obesity, diabetes, and inflammatory responses. Furthermore, epitranscriptomics modifications are dynamic processes; therefore, manipulating modifications could lead to tumor reversion, offering a promising therapeutic strategy [98]. In conclusion, an improved understanding of RNA modifications in cancer may contribute to the development of new diagnostic and therapeutic strategies for RCC.

### Reporting summary

Further information on research design is available in the Nature Research Reporting Summary linked to this article.

### Supplementary information

Reporting Summary
